# Enhancing mHealth data collection applications with sensing capabilities

**DOI:** 10.3389/fpubh.2022.926234

**Published:** 2022-09-15

**Authors:** Maximilian Karthan, Robin Martin, Felix Holl, Walter Swoboda, Hans A. Kestler, Rüdiger Pryss, Johannes Schobel

**Affiliations:** ^1^DigiHealth Institute, Neu-Ulm University of Applied Sciences, Neu-Ulm, Germany; ^2^Institute of Clinical Epidemiology and Biometry, University of Würzburg, Würzburg, Germany; ^3^Institute of Medical Systems Biology, Ulm University, Ulm, Germany; ^4^Institute for Medical Information Processing, Biometry and Epidemiology, Ludwig Maximilian University of Munich, Munich, Germany

**Keywords:** sensors, mHealth, software architecture (SA), mobile data collection, smart mobile devices

## Abstract

Smart mobile devices such as smartphones or tablets have become an important factor for collecting data in complex health scenarios (e.g., psychological studies, medical trials), and are more and more replacing traditional pen-and-paper instruments. However, simply digitizing such instruments does not yet realize the full potential of mobile devices: most modern smartphones have a variety of different sensor technologies (e.g., microphone, GPS data, camera, ...) that can also provide valuable data and potentially valuable insights for the medical purpose or the researcher. In this context, a significant development effort is required to integrate sensing capabilities into (existing) data collection applications. Developers may have to deal with platform-specific peculiarities (e.g., Android vs. iOS) or proprietary sensor data formats, resulting in unnecessary development effort to support researchers with such digital solutions. Therefore, a cross-platform mobile data collection framework has been developed to extend existing data collection applications with sensor capabilities and address the aforementioned challenges in the process. This framework will enable researchers to collect additional information from participants and environment, increasing the amount of data collected and drawing new insights from existing data.

## 1. Introduction

Collecting data in various scenarios has become an important factor for researchers and healthcare providers from different fields. Especially in medical or psychological scenarios, a lot of personal health data is needed to provide appropriate treatment to patients. Such data, in turn, are predominantly collected with pen-and-paper questionnaires (so-called *instruments*), which have a variety of drawbacks. The collected data must be transferred into digital spreadsheet documents, which is error-prone and time-consuming. Especially in large-scale and long-lasting scenarios such as clinical trials, this approach is outdated in most cases. For example, Pavlović et al. (1) found that about 50% of the cost of data entry can be saved if digital instruments are used instead of traditional paper-based instruments. In addition, studies show that digitally captured data sets have a higher degree of completeness (2) and higher data quality (3). Most importantly, participants accept such digital solutions (4, 5), and psychometric properties (6) are not violated. Recent publications also show an increasing use of electrocardiography sensors in conjunction with smart mobile devices to diagnose various diseases (7–14). Sensors are also used to measure heart rate variability (HRV). For example, recent literature describes an interplay between HRV and various psychological and physiological diseases (15–17).

In the last decade, smart mobile devices (i.e., smartphones or tablets) have become a ubiquitous part of our daily lives. In this sense, such devices can provide researchers with new opportunities to collect large amounts of big data in a relatively short period of time (18). In addition, mobile devices are equipped with many sensors (e.g., microphone, GPS, camera), which allows additional opportunities for metadata collection. For example, researchers can now not only provide digital questionnaires to assess participant data (i.e., patient-related data), but also collect environmental (e.g., current location) or physiological data (e.g., pulse measurement while working on the instrument). This is often referred to as remote measurement technology (19). In the context of this manuscript, we consider anything that generates data automatically (i.e., without human interaction) to be *sensor*. For example, internal hardware sensors such as microphone, GPS, or battery status, but also software applications such as the internal clock or external web services (e.g., for weather information) should be mentioned at this point. Unfortunately, there is no standard operating system for mobile devices. The market share of operating systems for mobile devices is mainly divided between iOS and Android. As a direct result, mobile application development can be cumbersome, especially when targeting an audience from both platforms (i.e., iOS and Android). When developing *native* applications for these platforms, the latter in turn enforces the use of the vendors' intended programming languages (i.e., Java or Kotlin for Android; Objective C or Swift for iOS), software patterns and paradigms, and platform-specific APIs.

To cope with the complexity of native application development, the so-called cross-platform development frameworks aim to take an alternative development approach. In this approach, a single code base is used to create a mobile application for different platforms (20). Most frameworks rely on modern web technologies to achieve the latter. Unlike native applications, such web-driven applications are not tied to a specific operating system, but to a specific browser implementation that is largely standardized. Schobel et al. (21) describes and compares different implementation strategies and approaches for such mobile cross-platform applications. Thus, developing mobile data collection applications using such cross-development approaches can also be a viable strategy, as such applications mainly consist of simple form elements for data entry (22). Moreover, the resulting application is readily available for all major platforms and devices. To date, there is no generic approach for integrating sensors into sophisticated data collection scenarios, such as healthcare. However, sensor frameworks that bundle commonly used sensors or offer different communication protocols (i.e., common wireless standards) are predominantly developed for native applications (i.e., Android or iOS platforms).

To support researchers in sophisticated data acquisition in practice, a sophisticated sensor framework is required for the reasons mentioned above. The latter must be compatible with the cross-platform development approaches to significantly accelerate development. Finally, the framework should allow application- and scenario-specific adaptations to meet researchers' requirements.

In the course of this manuscript, we mainly focused on the following research questions:

**RQ1:** Is it possible to realize a pluggable sensor framework with state-of-the-art cross-platform development technologies?
**RQ2:** How can we provide mechanisms that allow IT experts to easily integrate our developed framework into their own existing mobile data collection application?
**RQ3:** How can we develop and maintain an easy to extend framework, allowing to deal with demanding requirements from researchers when collecting data from a multitude of sensors?

The main contributions of the manuscript are as follows:

- We describe different *Application Scenarios*, where sensors can be used to extend the patient-reported datasets. Furthermore, we present *Requirements* derived from long-running real-world scenarios.
- We present a generic extensible framework *Architecture* to extend already existing mHealth data collection applications with sensing capabilities.
- We illustrate different *Sensor Interaction Patterns* that describe the communication between Sensors and the mHealth data collection application.

## 2. Related work

Several projects have already been proposed in research and industry, and a kind of sensor frameworks have been proposed and implemented by them. In this section, related work are discussed in this context.

The authors of Katevas et al. (23) present a framework that enables the communication with and the collection of data from many different sensors. For this purpose, the *SensingKit* framework provides special client libraries, both for the iOS and Android platforms. These libraries can be integrated into an existing mobile application to support a wide variety of on-device sensors, such as the microphone, GPS sensors or the camera. External sensors are controlled *via* wireless communication using Bluetooth Low Energy (24). In Ferreira et al. (25), the authors present AWARE, a framework for mobile context instrumentation. AWARE provides a ready-to-use client application for Android and iOS to collect sensor data and information (e.g., free text such as opinions, sentiments, and others). This framework enables researchers to create mobile data collection applications with sensor capabilities, but without the requirement of programming skills. In addition, the framework is available as an Android and iOS library for developers to use in their own applications. Similar to the aforementioned frameworks, Brunette et al. (26) presents a sensor framework (Open Data Kit), capable of connecting many different sensors. However, *Open Data Kit* is only available for Android, which limits its applicability in large-scale research projects. All three projects cannot be used in the development of applications if cross-platform development approaches are pursued.

RADAR-Base is an open source mobile health platform for collecting, monitoring and analyzing data using sensors, wearables and mobile devices (27). RADAR-Base provides a full-featured platform with an application for passive and active monitoring, an Apache Kafka-based backend, and a management portal for configuring pilot studies. The project has been successfully applied in various scenarios such as depression, multiple sclerosis, epilepsy, and Alzheimer's disease (28, 29). While the platform provides a sophisticated all-in-one solution for data collection, monitoring and analysis, there are scenarios where the development of a new mobile application is required and the use of a pure sensor framework that simplifies the use of sensors is preferable.

There are several other platforms such as RADAR-Base and AWARE, all of which focus on a user-friendly approach that allows researchers to easily create studies, deploy them in a generic mobile application, and store the collected data in the cloud [e.g., LAMP (30), Sensus (22), mCerebrum (31)]. However, medical or psychological studies often have challenging requirements for which such generic "one-size-fits-all" solutions may not be suitable. In such scenarios, the proposed sensor framework takes a different approach that offers valuable advantages. In particular, it allows developers to create custom-tailored mobile applications and extend them with sensing capabilities. In addition, it allows researchers to store collected data on-site rather than in the cloud, which may be of particular interest in the context of medical psychological studies (i.e., GDPR concerns).

*Google Fit* is a cloud-based platform for collecting and storing fitness and health data, developed and provided by Google^1^. The platform allows developers to collect data from a mobile application and store and share data from the device's sensors in a central hub. External sensors (e.g., a heart rate monitor) are connected *via* Bluetooth Low Energy if the required standard GATT profile is available. Google provides an extensive API for communicating with sensors. Common sensor data types are pre-modeled, allowing IT professionals to develop their applications in a relatively short time. In line with this, Google provides a full-fledged Android library for fitness application development. Mobile applications developed for other operating systems (e.g., iOS) rely on a RESTful API to communicate with the server. RESTful APIs are a common architectural style and development paradigm for web services (32) .

Similar to Google Fit, Apple also offers its *HealthKit* platform^2^ with similar functionality. However, access to this platform and the corresponding data is only possible *via* the Apple ecosystem using special software development kits that are only provided for iOS.

Henriksena et al. (33) describes an approach to using Google Fit and Apple HealthKit to collect fitness data in a large-scale population study. This data will in turn provide detailed insight into participants' physical activity. Researchers expect great benefits from accessing such health data stores, as they may also contain historical information. Farshchian and Vilarinho (34) compares the featured platforms by developing a health application. The researchers also discuss performance issues of sensors between different smart mobile devices when developing cross-platform applications (35). Note, however, that depending on the device manufacturer, the quality of sensors (i.e., resolution, Frequency) may vary. This potential issue may not be a problem with Apple devices, but can be critical when developing for the Android platform.

## 3. Concept

In this section, a running application scenario for this manuscript is presented and requirements are derived from it. Finally, an extensible architecture for the sensor framework is presented.

### 3.1. Application scenarios

The use of sensors to capture additional information during data acquisition has become commonplace in a variety of application areas. When developing the concept and proof-of-concept implementation of the present sensor framework, we had two different application scenarios from the medical domain in mind. These scenarios are explained in more detail below.

#### 3.1.1. Remote patient monitoring

According to the *World Population Aging Report* (36), the number of elderly people increased substantially within recent years, with about 900 million people worldwide aged 60 years or above in 2015. This trend seems to go even further, with an estimated increase of 50% until 2030 (36). Furthermore, the number of people suffering from chronic diseases, such as heart failure or diabetes, increases dramatically (37). With an increasing number of people requiring long-term medical treatment, traditional healthcare approaches (i.e., on-sight patient examination and treatment) could easily reach their limits. Therefore, a shift toward delivering remote healthcare may significantly relieve healthcare systems and provide benefits for patients. As a direct consequence, one central research topic focuses on the design and implementation of applications enabling a continuous monitoring of the health status outside a clinical environment.

For example, Bot et al. (38) evaluated the feasibility of remotely collecting information about changes in symptoms of patients with Parkinson's disease. With the current standard of care, affected patients would visit a physician every 4−6 months. The approach proposed by the authors requires patients to participate in self-assessments daily *via* a provided mobile application. Besides processing Parkinson-specific questionnaires, participants were asked to execute physical tasks on a day-to-day basis. These tasks incorporate smartphone sensors, such as microphones for recording voice activities or accelerometers, and gyroscopes for evaluating the patients' gait and balance.

In Suh et al. (39), a platform for monitoring patients with congestive heart failure is presented. The respective platform aims to facilitate the early detection of acute symptoms, prevention, monitoring, and treatment of such patients. The developed application can communicate directly with external sensors in weight scales or blood pressure monitors *via* Bluetooth. Further, smartphone sensors are combined to implement fall detection. A real-world study showed that the number of weight and blood pressure measurements outside of an acceptable range could be successfully reduced with the proper use of such an application.

Besides monitoring patients with chronic diseases, remote patient monitoring may also be applied in other medical scenarios. Marko et al. (40) investigated the applicability of mobile applications with other connected devices for monitoring health conditions in prenatal care scenarios. Again, in this setting, digital weight scales and blood pressure cuffs were given to participants to collect data at home during pregnancy. Collected data were scanned for irregularities and clinicians were informed. An associated study revealed a high patient satisfaction and was able to identify episodes of abnormal weight gain. Due to the COVID-19 pandemic, there was an increased need to monitor the health status of patients outside of the hospital to prevent the spread of the virus. Yamamoto et al. (41) investigated how a mobile application for personal health records (PHR) could be used to accomplish this task. They found that health observation with PHRs can also be used effectively as a measure against infectious diseases.

#### 3.1.2. Intensive longitudinal methods

Intensive longitudinal methods summarize various research methodologies, such as *Experience Sampling, Daily Diaries*, or *Ecological Momentary Assessments* (EMA) (42). The named methods may be used to examine feelings, thoughts, or behaviors in a natural, real-time context and frequently (i.e., daily) over an extensive period.

The *TrackYourTinnitus* platform, for example, relies on Ecological Momentary Assessments to support researchers in collecting data from patients suffering from tinnitus (43). Since tinnitus is a subjective perception, assessing symptoms can only be achieved with the help of reports from affected patients (44). In addition to a digital data collection procedure, TrackYourTinnitus uses smartphone-internal sensing capabilities to enrich the dataset with contextual data, like the current GPS position or environmental sound level.Most importantly, the TrackYourTinnitus platform and its generic API (45) have been adapted to other diseases as well. For example, the same technology stack is used to support researchers in assessing data in the context of stress (46), diabetes (47) or hearing loss. The study presented in Beierle et al. (48) examines physical and mental well-being during the COVID-19 pandemic using an app that combines questionnaire-based surveys with mobile sensor recordings.

The study described in Cao et al. (49) investigated the feasibility of using smart mobile devices to monitor depression symptoms. Among typical self-reported data, the mobile application collected a variety of different sensor data regarding the movement (step counter, GPS coordinates) or social interaction (amount of messages, call duration). Study results indicate that the combination of data was able to predict the score of *PHQ-9* (50), a well-established and validated instrument from clinical psychology, with an accuracy of 88%. Additional information provided by relatives of patients further increased the accuracy, allowing researchers to predict the respective outcome. Schobel et al. (51) evaluated a mobile application to support medical staff from psycho-oncology through mobile data collection. Comparing the acceptance of mobile digital screenings with paper-based ones. Sixty participants were divided into two groups, 31 of which used paper-based questionnaires and 29 the digital version. The results show that the general acceptance increases by 58.5% in the mean when the digital instead of the paper-based approach is used.

### 3.2. Challenges and requirements

As described in Section 3.1, gathering sensor data may be indispensable in mobile data collection scenarios, especially in healthcare. Integrating sensors into the data collection procedure may result in several benefits for both, study directors (i.e., researchers, medical staff) and participants (i.e., patients).

Smart mobile devices offer a huge variety of internal sensing capabilities and interfaces to connect with external sensing devices. However, there is no uniform way of addressing such sensors generically. Sensors differ in their type of connection (i.e., internal, wired or wireless), communication protocols (i.e., direct API, Bluetooth), interaction paradigms to collect data, or their output format, just to mention a few factors. Depending on the respective mobile operating system (iOS vs. Android), communicating with such sensors requires specific APIs. This requirement imposes massive challenges for IT experts as it requires deep knowledge about all the different sensors, the underlying platform infrastructure, and the application domain, respectively. Gathering knowledge about the API of each sensor which the IT expert must use in an application is a time consuming task. A sensor framework should reduce the time required by offering an abstraction layer over all used sensors. The saved up time can then be invested in developing the actual mHealth application instead of reading API documentations for every sensor.

Existing mobile applications used for data collection purposes may already access sensor information to enrich participant data. In this context, they often use dedicated, application-specific implementations. However, this results in hard-to-maintain applications or reuse existing features in other applications or scenarios. While there are libraries and frameworks (see Section 2) that aim to provide a more generic and standardized way of addressing a broad spectrum of available sensors, the latter may only be available for specific platforms or lack the functionality required for more complex scenarios. To support IT experts in developing sophisticated applications using cross-platform development strategies, a novel framework was realized. In this context, further requirements were extracted from conducting structured interviews with experts responsible for collecting data in different medical projects. Further, insights from implementing long-running mobile data collection applications and deploying the latter in real-world scenarios were considered. The insights from implementing the projects shown in Table 1 were incorporated into the requirements. Finally, requirements from the literature were added as well.

**Req-01 (Support Device Internal Sensors):** Modern smart mobile devices are equipped with a rich set of onboard sensing capabilities. The framework should provide ways to access and communicate with these sensors and gather data from them.
**Req-02 (Support External Sensors and Devices):** Smart mobile devices offer a variety of interfaces to communicate with external resources *via* different protocols. The connection may be wired (i.e., USB) or wireless (i.e., WiFi, Bluetooth). The framework should therefore provide possibilities to establish a connection with such devices.
**Req-03 (Provide Default Set of Sensor Implementations):** The framework should provide a set of default sensor implementations that can be used in common application scenarios.
**Req-04 (Custom Sensor Implementations):** Since there are many different sensors, it is impossible to provide predefined implementations for every single one. Therefore, the framework should allow for communicating with framework-compliant sensor implementations.
**Req-05 (Fine-grained Configuration during Runtime):** Sensors differ in the type of data they measure and their behavior (i.e., sampling rate, resolution). This configuration should be customizable when requesting data during runtime, allowing for a more versatile framework use in different application scenarios.
**Req-06 (Support Different Sensor Interaction Patterns):** Sensors differ in the type of data they provide and how they are providing it (i.e., provide a single measurement vs. continuously sending data). The framework should support a variety of interaction patterns for various sensors.
**Req-07 (Offline Usage):** Depending on the application scenario or the application's environment, a stable Internet connection may not be guaranteed. The framework should follow an *offline-first* approach. Sensor implementations should not require an Internet connection in general.
**Req-08 (Support Different Mobile Operating Systems):** Existing frameworks often target one specific mobile operating system. The framework described in this manuscript explicitly focuses on a cross-platform approach, resulting in a framework that can be used for both iOS and Android applications.
**Req-09 (Output Format):** Data gathered from sensors should be described and formatted to be suitable for further processing (i.e., visualization). Sensors, therefore, should provide respective meta- information about themselves and their data.
**Req-10 (Fault Tolerance):** By providing a generic way of communicating and accessing sensors on different platforms, specific error scenarios have to be taken into account (i.e., sensors are not available, hardware failure). Errors in the framework should not cause the host application to stop working or even crash.
**Req-11 (Extensibility):** It should be easy to extend the functionality of the framework by adding more custom sensors. The framework itself should favor a plug-and-play architecture.
**Req-12 (Easy Integration of the Framework):** Since the primary purpose of the framework is to enhance existing mobile data collection applications with sensing capabilities, integrating the latter should be as easy as possible.

**Table 1:** Realized mHealth data collection applications.

**mHealth application scenario**	**Country**	**Sensors**
Study on tinnitus research (52)	World-Wide	Microphone, GPS
PTSD in war regions (53)	Burundi	Microphone, Camera
PTSD in war regions (54)	Uganda	Microphone, Camera
Adverse childhood experiences (55)	Germany	Microphone, Camera
Learning deficits among medical students	Germany	Pulse
Corona check (56)	Germany	GPS
Corona health (48)	Germany	GPS, Apps used

PTSD, post-traumatic stress disorder.

### 3.3. Architecture

**Figure 1:**
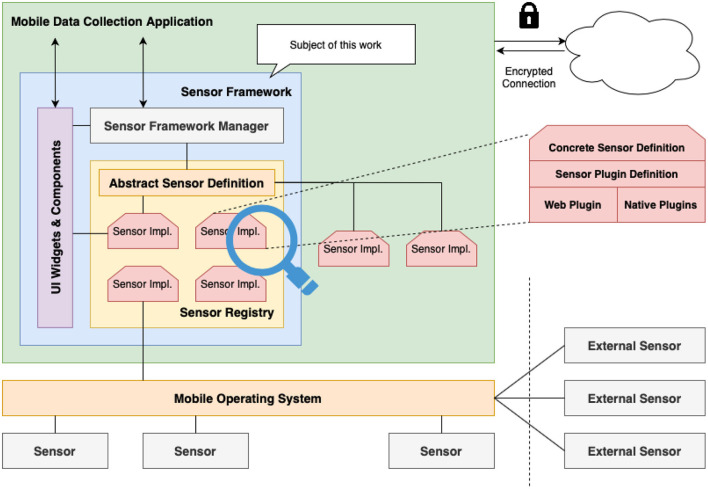
Architecture of the sensor framework.

When carefully elaborating the requirements, a modular and extensible software architecture was developed (see Figure 1). The framework itself should be a ready-to-use module comprising all necessities and features to be integrated into an existing mobile data collection application. Communication between the data collection application and sensors should not take place directly, as this may lead to unwanted side effects. Instead, the communication should be routed through a central communication and coordination unit within the sensor framework (see *Sensor Framework Manager*, Figure 1), which provides an extensive interface for the data collection application.

To address specific implementations in a generic way, proper abstraction mechanisms were implemented and made available (see *Abstract Sensor Definition*). The latter defines basic interfaces and already implements common behavior for sensors within the framework. This abstraction can also be used when implementing custom sensor drivers, which may be specifically tailored for a given application scenario. While this abstract definition provides a solid foundation for all sensors, the latter can be refined with sensor-specific implementations (see *Concrete Sensor Definition*) to better reflect specific behavior or available interaction patterns. The communication with the actual sensor for both internal and external devices, is implemented in dedicated native implementations (see *Web* and *Native Plugins*).

All sensor implementations to be discovered and accessed through the framework are registered in a central *Sensor Registry*. The latter may include some predefined sensors shipped with the framework (i.e., microphone, GPS, camera) as well as custom sensor implementations defined outside the framework.

Finally, the framework provides a set of user interface components (see *UI Widgets*) that can be embedded directly into an existing UI of the data collection application. Notably, custom sensor implementations may also provide their own visualization.

This modular design guarantees that different parts of the framework can be easily adjusted or extended according to scenario-specific needs.

Note the work presented in this manuscript does not cover the architecture and implementation of the mobile data collection application nor the backend service that can store all collected information.

## 4. Implementation

This section of the manuscript provides in-depth information on the implementation of the actual sensor framework. The sensor framework was developed using an agile software development process (57). New features were derived from user stories and developed in sprints over 2 weeks. A sprint was typically dedicated to one user story and went through the following steps: planning, designing, developing, testing, and reviewing.

Since one of our main requirements is that the framework should be used across different platforms, we decided to rely on the well-established *Ionic* framework (Version 5.0.0)^3^. The latter allowed us to build the framework using state-of-the-art web technologies. Further, the framework can be executed within a web browser, but also enabled us to integrate the framework into mobile applications built with *Capacitor* (Version 1.4). Capacitor, in turn, is a runtime environment that allows accessing the native platform features and APIs. As another feature of Capacitor, IT experts are not tied to a specific frontend framework to represent the user interface. Our prototype implementation relies on a lightweight web components framework called *Stencil*^4^. Those components can be easily integrated into the latest state-of-the-art frontend frameworks like Angular, React, or pure Vanilla JavaScript. The source code of the sensor framework can be found on Github^5^.

### 4.1. Sensor communication

Due to its energy efficiency, Bluetooth Low Energy enjoys growing popularity as a wireless communication standard for external sensing devices (i.e., heart rate monitors or weight scales). In order to be able to communicate with such external devices, a respective Bluetooth connector was implemented, which is compatible with all peripheral devices implementing one of the standard Bluetooth profiles. The connector allows for scanning for available devices, connecting and disconnecting a sensor to the mobile device or requesting data from the sensor. Behind the facade, respective native APIs from Android or iOS are accessed. Furthermore, web-based implementations are provided to be able to use the framework in browser-based environments as well. Moreover, a common interface for internal sensors (i.e., camera, GPS) was implemented. Again, this class acts as an abstraction layer to platform-specific native implementations.

### 4.2. Sensor interaction patterns

**Figure 2:**
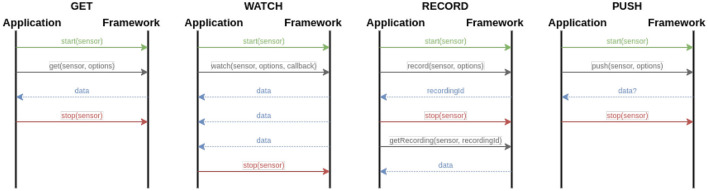
Identified and implemented sensor interaction patterns.

When analyzing existing data collection applications that use sensors to collect additional information from participants, we were able to identify different interaction patterns between the involved parties (i.e., sensor and mobile application). These patterns are illustrated in Figure 2 and described in more detail below.

**GET:** When using this pattern, the sensor returns a single measurement (i.e., get the current location of the participant).
**WATCH:** This pattern returns a continuous stream of sensor measurements (i.e., when monitoring the heart rate of a patient). A callback method can be passed, which is triggered for each measurement.
**RECORD:** This pattern allows for gathering sensor data until the sensor is stopped (i.e., record a video or audio stream). When calling the record() method, a recordingId is returned. Data can be retrieved later *via* this Id, once the sensor is stopped.
**PUSH:** This pattern allows for pushing data from the application to the sensor (i.e., making a HTTP POST request).

All aforementioned methods used to interact with the sensor accept an optional options parameter. This parameter is specific to one particular sensor and may be used to configure its behavior during runtime (i.e., change the camera's resolution, use kg instead of lb). Note that the sensors have to implement a respective hook method to be able to take part in such an interaction pattern (i.e., implement the onGet(), onWatch(), onRecord() or onPush() methods).

## 5. Integration

This section finally illustrates the process of enhancing an existing mobile application with sensing capabilities, as well as the workflow to extend the framework with custom sensors. It should be mentioned that data privacy is outside the scope of the implemented framework. Therefore, the developers of the mobile application which uses this framework are in charge ensuring the privacy of user data. As the main target of most applications with sensing capabilities is data collection, it can be assumed that those applications will use some sort of backend to store the collected data. In this case, the developers have to ensure that the connection between the application and the backend is encrypted and that the stored data is safe concerning the users' privacy. There are some actions to improve privacy on the operating system level (e.g., iOS and Android require user permission to access certain internal sensors), but those are not sufficient to guarantee data privacy for the whole system.

### 5.1. Enhancing existing applications with sensing capabilities

The proposed sensor framework is meant to be used to enhance existing applications. As such, it could be, for example, used to add remote measurement technology to an mHealth application. For demonstration purposes, a new mobile application was created. The respective sensor framework that was introduced in the course of this manuscript can be easily added as a dependency *via* common package manager, like npm or yarn. As the user interface of the developed application relies on web components (i.e., *via* Stencil), the automatically generated defineCustomElements() utility method has to be called at the top-most level of the application hierarchy^6^. Note that other state-of-the-art frameworks like React or Vue can also be used as they adhere to the web components standard.

In general, there are two different ways of interacting with the framework during run time. Two Angular components were implemented to showcase both approaches, each of them covering one particular approach of interacting with a different sensor.

#### 5.1.1. Access sensor *via* sensor framework manager

The more common approach of accessing sensor data is by directly addressing the provided Sensor Framework Manager. For example, we implemented a component for displaying the current location of the user. Thereby, the position should be initially retrieved from the GPS sensor and updated continuously whenever the location of the device changes.

After importing the Sensor Framework Manager instance in our application, the latter can be used to access the GPS capabilities of the devices directly. Note that the framework already provides a ready-to-use implementation for this sensor. This predefined implementation may also serve as a blueprint for custom adaptations if needed. The device's current position can be requested by calling the get() method (see Section 4.2). In order to continuously receive updates on the location, the watch() method should be called. This method, in turn, triggers a user-defined callback method, which can be used to adjust the map displaying the current user location properly.

#### 5.1.2. Access sensor *via* web component

The second approach offers a higher level of abstraction by making use of the provided HTMLSensorElement. To showcase the HTMLSensorElement approach, a Bluetooth Low Energy heart rate monitor is connected. The retrieved data, in turn, is visualized through a new user interface widget (see Listing 1).

**Listing 1:**
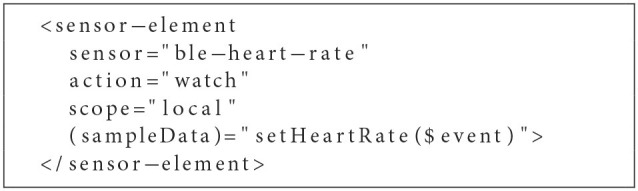
Custom HTMLSensorElement within heart-rate.component.html.

As indicated, the sensor can be set up by simply integrating the HTMLSensorElement within the HeartRateComponents template. Further, sampleData events containing collected sensor data may be intercepted by binding the event to a corresponding handler within the business logic of the HeartRateComponent (see Listing 2).

**Listing 2:**
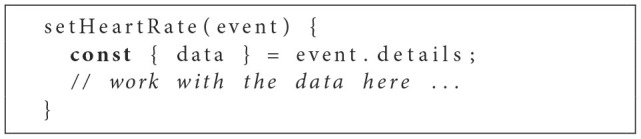
Event Handler for Heart Rate Measurements.

This template-based approach of accessing sensors and their respective data is beneficial to dynamically gather data from multiple sources at once (i.e., when building some kind of dashboard or aggregating data from different sensors). As can be seen below, multiple HTMLSensorElements may be created by looping over an array of corresponding configuration objects. Emitted data can then be aggregated and processed by binding events thrown by the sensor to a common event handler (see Listing 3).

**Listing 3:**
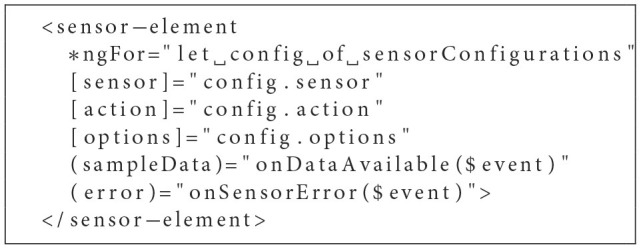
Dynamic Creation of multiple HTMLSensorElements.

### 5.2. Extending the framework with custom sensors

To demonstrate how to extend the developed framework with additional sensing capabilities, a new sensor was implemented and added to the previously described demo application. This sensor, in turn, monitors the battery level of the device.

First, a CustomBatterySensor class, extending the already existing Sensor base class, has to be created. Note that there also exist other base classes to extend from (i.e., the BluetoothSensor) that may offer more features required (i.e., establishing a connection). Next, respective *sensor interaction patterns* (i.e., get() and watch()), as well as a suitable configuration for this sensor, are defined and implemented. The business logic of the sensor itself holds a reference to the Battery Manager from the underlying platform. Note that this manager is also exposed and accessible through common web browser interfaces. The onGet() method may query the current battery level, whereas the onWatch() method triggers a callback when the charging state changes (i.e., from *normal* to *charging*).

**Figure 3:**
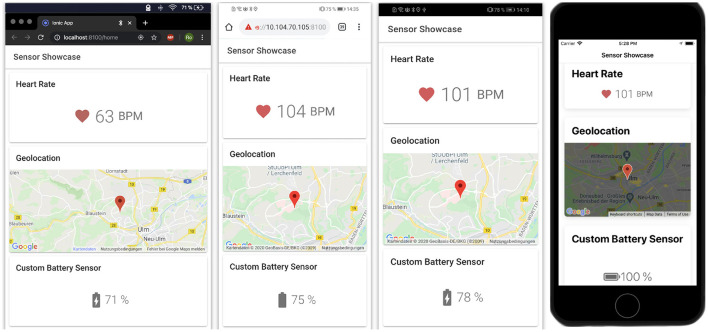
Resulting application running on different platforms. From left to right: Web Application on Google Chrome, Web Application on Chrome for Android, Native Android Application, and Native iOS Application.

The resulting application with its three implemented sensors (GPS, heart rate and battery) running on different platforms is illustrated in Figure 3. Note that the user interface looks the same across all platforms, but can be easily customized to fit platform-specific guidelines if needed. Notably, the application is developed once and can be deployed and used on all major platforms.

Our sensor framework also includes a set of predefined implementations to be used in different application scenarios. The latter include ready-to-use classes for addressing device-internal sensors as well as external sensing devices. Table 2 provides a brief overview of implementations on different platforms and current web browsers. Note that the availability of built-in sensors (and connectivity interfaces like ”Bluetooth Low Energy”) may differ.

**Table 2:**
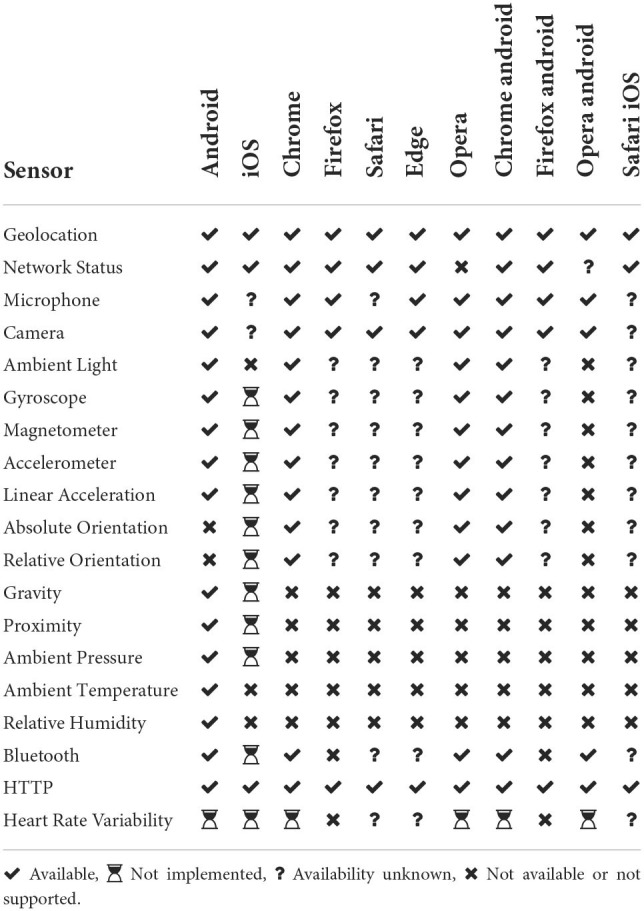
Availability for predefined sensor implementations on different platforms.

## 6. Limitations

The limitations of this work are on the one hand technical and on the other hand related to the choices made in the construction of the framework. Therefore, the various limitations are presented in the following two categories.

### 6.1. Technical limitations

In order to be platform-agnostic, the entire framework was developed based on web technologies, or more precisely, it was built on Capacitor. This leads to the fact that the framework can be integrated into mobile applications on different platforms, but this also brings disadvantages. Since native access to the mobile sensor APIs is provided by dedicated Capacitor plugins, the framework is tightly coupled with this ecosystem. Therefore, the framework is only suitable for use in regular web applications or mobile applications that are based on the Capacitor runtime environment.

Another limitation arises when the framework is to be integrated into web applications. Since the variety of different browsers is much larger than for mobile operating systems, it cannot be assumed that all browsers in different versions support the necessary functions to address mobile sensors. For example, some APIs used by the framework to address sensors *via* the web browser are marked as experimental functions that must be manually need to be enabled in the browser settings (e.g., SensorAPI or Web Bluetooth API). To realize the full potential of the developed framework, broader support of modern web APIs in all browsers is required.

Web API implementations in different browsers also need to become more reliable. For example, during the development of the framework, a browser update caused the *NetworkStatusSensor* returned inaccurate data. Such a problem could be prohibitive in production scenarios. However, the latter issues only affect the web version of the corresponding sensor implementations, not the native ones.

Although setting up the framework within an existing application is relatively easy, a complete “plug-and-play” solution could not be achieved. The setup requires some manual configuration steps where some lines of code had to be added to the existing application. This mainly concerns the registration of the Capacitor Custom Native plugins in the existing application, which is tolerable but should not go unmentioned.

Since iOS 13, Apple has reduced the maximum time frame for background tasks to 30 s. There are ways to extend this time frame, but this is only possible for a limited number of allowed background tasks (e.g., location updates, audio playback, VoIP, use of Bluetooth LE accessories). Therefore, the application developer must ensure that the appropriate properties are set in the application. The Bluetooth LE accessory task may allow reading data from external sensors while the application is running in the background. Reading data from internal sensors is currently not possible in background mode. Another solution could be to use Internet-enabled external sensors that communicate their intent with a web service. The web service would trigger a push notification on the device. It is important to note that this is not part of the sensor framework, as these considerations have to be made for the respective iOS application itself.

### 6.2. Framework dependent limitations

A common limitation with frameworks is a certain restriction on developers' design freedom. We have tried to keep this to a minimum, but set specific guidelines that developers must adhere to. Furthermore, scenarios are conceivable in which the available sensor interaction patterns presented in Section 4.2 (GET, WATCH, RECORD, PUSH) are not sufficient. For these cases, the framework would have to be extended by further patterns.

## 7. Summary

In various application scenarios, data collection is becoming increasingly important for researchers. In some areas (e.g., healthcare), not only are questionnaires for self-assessment, but also data collected by sensors (e.g., to measure physiological parameters) important. As part of our research project, we evaluated different application scenarios where the benefit for researchers could be increased by integrating sensors. These findings, along with interviews with healthcare and psychology professionals, revealed important requirements for a generic sensor framework. A structured analysis of existing frameworks revealed that most of them are focused only on a specific platform (e.g., Android or iOS), are not flexible enough, or lack the functionality to be used in challenging real-world scenarios such as psychological studies or clinical trials.

As web technologies have matured, there has been a shift toward cross-platform development in industry and literature. To adapt to this, the sensor framework described in this manuscript is implemented using state-of-the-art web technologies. This approach allows IT professionals to easily integrate it into any web-based mobile application (i.e., developed using Ionic) or into a browser-based applications (e.g., developed with Angular or Electron).

This manuscript illustrates the design process for a sophisticated sensor framework in a reproducible manner. In addition, key aspects such as extensibility are highlighted in detail so that others can accurately understand these requirements. By elaborating various use cases for the application of such a framework, we were able to extract different sensor interaction patterns. The realized sensor framework and its extensible approach provide a solid foundation for future mobile data collection applications in a variety of scenarios.

However, further investigation is required as part of this project. Among other things, the framework is to be used in a research project in the field of psycho-oncology in order to improve the possibilities of data collection here. Interviews with IT experts working on this project could in turn reveal further interaction patterns or additional requirements. This may lead to adding more sensors to the framework. It may also be possible to develop a special library containing mainly sensors. Such a library can also be added *via* package managers. Also, additional support for connectivity aspects may be added over time. Currently, WiFi and Bluetooth are implemented, but USB will be added gradually for native platforms. Web applications, in turn, could make use of the WebUSB API available in modern browsers. Another useful extension would be the integration of other services such as the *Google Fit REST API* to integrate functions offered by the respective services. Finally, the code should be made available as Open Source so that others can use, extend and improve it.

## Data availability statement

The source code of the developed sensor framework can be found at: https://github.com/hnu-digihealth/sensorframework.

## Author contributions

JS and MK analyzed the current as-is situation and conceived and designed the architecture and the prototype. RM and MK implemented the prototype. JS, MK, and RM mainly wrote the paper, whereas all authors gave input on the content. All authors contributed to the article and approved the submitted version.

## Conflict of interest

The authors declare that the research was conducted in the absence of any commercial or financial relationships that could be construed as a potential conflict of interest.

## Publisher's note

All claims expressed in this article are solely those of the authors and do not necessarily represent those of their affiliated organizations, or those of the publisher, the editors and the reviewers. Any product that may be evaluated in this article, or claim that may be made by its manufacturer, is not guaranteed or endorsed by the publisher.

## Footnotes

^1^https://developers.google.com/fit/overview; last accessed 2021-11-25.

^2^https://www.apple.com/ios/health/; last access 2021-11-25.

^3^https://ionicframework.com/; last accessed: 2021-11-29.

^4^https://stenciljs.com/; last accessed: 2021-11-29.

^5^https://github.com/hnu-digihealth/sensorframework; last accessed: 2022-01-14.

^6^The documentation can be found at https://stenciljs.com/docs/overview; last accessed: 2021-11-29.
